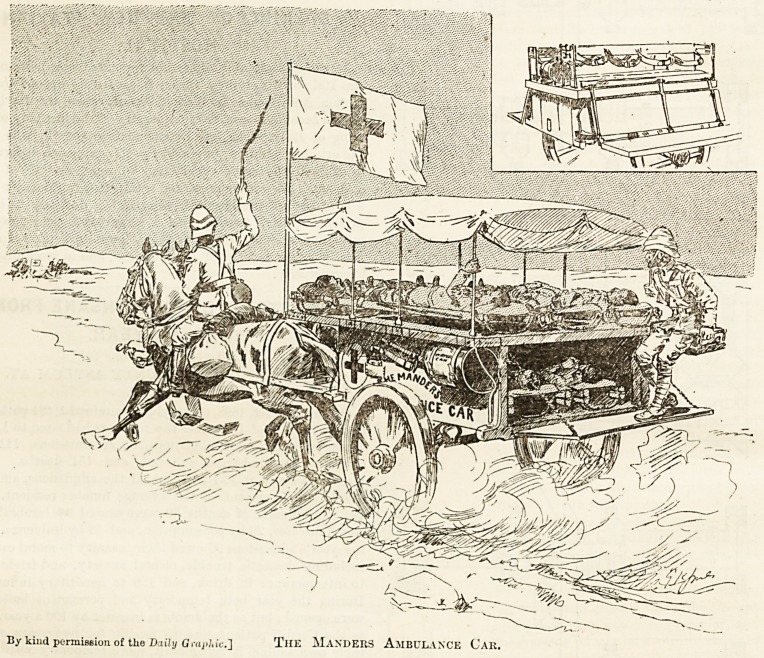# The Flying Hospital

**Published:** 1900-03-17

**Authors:** 


### THE FLYING HOSPITAL.

Dr. Manders has designed the two-wheeled cars for the wounded given in our illustration. The object of these cars is to secure rapid aid and comfort to the wounded on the field, and to mounted troops more especially. These cars can move as rapidly as artillery. The ambulance car consists of a well-ventilated body for two stretchers and some appliances, above which is the car platform to take four stretchers. The whole rests on carriage springs and has an awning roof. The hospital car is built after the fashion of an Irish car, with the centre boxed in, which serves as a receptacle for all requisites. Outside sit the attendants and also some of the wounded who can sit up. Each car is drawn by two horses, harnessed after a plan which provides for speed and mobility. The cars are an excellent arrangement to secure, firstly, immediate attention to the wounded on the field, and secondly, rapid and comfortable transport between the field and the nearest hospital. A committee has been formed to collect subscriptions to provide these cars for the Imperial Yeomanry. Two cars have been presented to the 12th Battalion, and the Coats family, of Ferguslie, have given two to the 6th Battalion. The cars can be seen at Messrs. Windover's, 22, Long Acre. The hon. secretary of the Flying Hospital Fund is Dr. Arthur James, 228, Gloucester Terrace, W.

By kind permission of the *Daily Graphic.*] The Manders Ambulance Car.

## Figures and Tables

**Figure f1:**